# Fracture of the Lumbar Spine Associated with Ureteral Injury Mimicking Spondylodiscitis Followed by Cervical Spine Fracture in Patient with Ankylosing Hyperostosis

**DOI:** 10.3390/jcm12216937

**Published:** 2023-11-05

**Authors:** Michał Woźnica, Szymon Kaczor, Łukasz A. Poniatowski, Mikołaj Raźniak, Mirosław Ząbek

**Affiliations:** 1 Department of Spine Surgery, 7th Navy Hospital, Polanki 117, 80-305 Gdańsk, Poland; michalwoznica.med@gmail.com; 2 Department of Neurosurgery, 1st Military Clinical Hospital in Lublin—Branch in Ełk, Tadeusza Kościuszki 30, 19-300 Ełk, Poland; kaczorszymon@gmail.com; 3 Department of Neurosurgery, Dietrich-Bonhoeffer-Klinikum, Salvador-Allende-Straße 30, 17036 Neubrandenburg, Germany; 4 Department of Neurosurgery, Mazovian Bródno Hospital, Kondratowicza 8, 03-242 Warsaw, Poland; razniak.mikolaj@poczta.onet.eu (M.R.); zabek.cmc@gmail.com (M.Z.); 5 Department of Neurosurgery, Centre of Postgraduate Medical Education, Kondratowicza 8, 03-242 Warsaw, Poland; 6 Interventional Neurotherapy Center, Mazovian Bródno Hospital, Kondratowicza 8, 03-242 Warsaw, Poland

**Keywords:** lumbar spine fracture, cervical spine fracture, ankylosing hyperostosis, ureteral injury, spondylodiscitis, osteomyelitis

## Abstract

The purpose of this case report is to describe the case of a patient with ankylosing spinal hyperostosis (ASH) and lumbar spine fracture complicated by ureteral injury mimicking spondylodiscitis with osteomyelitis features and retroperitoneal abscess formation followed by the cervical spine fracture. A consecutive analysis and summary of the medical history, radiological documentation, operative procedure, complications, and outcomes were performed. A 59-year-old man presented with abdominal pain three weeks after sustaining a low-energy fall. The performed CT scans demonstrated a three-column fracture at the L3/L4 level and features of ASH. Additionally, MRI scans demonstrated hyperintense fluid collection within L3/L4 intervertebral space communicating with both psoas major muscles, mimicking spondylodiscitis with osteomyelitis features and retroperitoneal abscess formation. An in situ instrumented lumbar fusion at the L2-L3-L5-S1 levels with implantation vertebral body replacement implant at the L3/L4 level was performed. Postoperative CT imaging revealed evidence of post-traumatic right ureteral injury. Following urological treatment covering nephrectomy and ureter ligation, the patient was maintained at a 2-year follow-up. After this period, the patient presented again with tetraparesis after sustaining a low-energy fall. The performed CT scans demonstrated a three-column fracture at the C5/C6 level. The combined anterior and posterior osteosynthesis at the C4-C5-C6-C7 levels was performed. This case report presents the rare clinical constellation regarding the lumbar spine fracture complicated by ureteral injury followed by a cervical spine fracture regarding the same patient. The potential injury of retroperitoneal structures, including the ureter after hyperextensive lumbar spine fracture, should be considered in ASH patients. In this case, one should be aware of the atypical clinical presentation regarding the observed spondylodiscitis- and osteomyelitis-like features.

## 1. Introduction

The management and therapy of vertebral column fractures in patients with ankylosing spinal hyperostosis (ASH) or diffuse idiopathic skeletal hyperostosis (DISH) pose a clinical challenge due to the pre-existence of long, calcified, and fused vertebral segments as well as its associated biomechanical alternation of spinal column [1,2]. In this case, vertebral column fractures are often associated with instability, where patients are more likely to exhibit neurologic deficits and undergo operative procedures with higher complication and mortality rates [3]. Therefore, the general complication rate in this patient population is estimated as 32.7%, while overall mortality within 3 months after injury is estimated as 20.0% [4]. Among all genitourinary injuries, ureteral trauma constitutes a rare and uncommon condition, accounting for less than 1% of all blunt and 4% of all penetrating injuries [5]. Collectively, the penetrating injury of the ureter as a result of the vertebral column fractures constitutes an unusual complication with a statistical rate estimated at 0.3% [6]. Nevertheless, we were unable to find precise statistical data on this issue. To the best of our knowledge, only two cases of patients with lumbar fractures and associated ureteral injury have been reported [7,8]. Given this evidence, we intend to report and discuss significant features of this rare and extraordinary case of a patient with ASH and unstable lumbar fracture at L3/L4 level complicated by ureteral injury mimicking spondylodiscitis with osteomyelitis features and massive retroperitoneal abscess formation. This study contains a literature review to provide a comprehensive information repository for spine surgeons regarding this complication and its management options. Moreover, after a 2-year follow-up post-initial trauma, the same patient was presented to the hospital due to an unstable cervical fracture at the C5/C6 level with tetraparesis. However, according to the advanced literature search, there have been no academic publications regarding a case with such a clinical constellation to this day.

## 2. Case Report

### 2.1. Clinical Presentation during the First Treatment Episode

**Figure 1:**
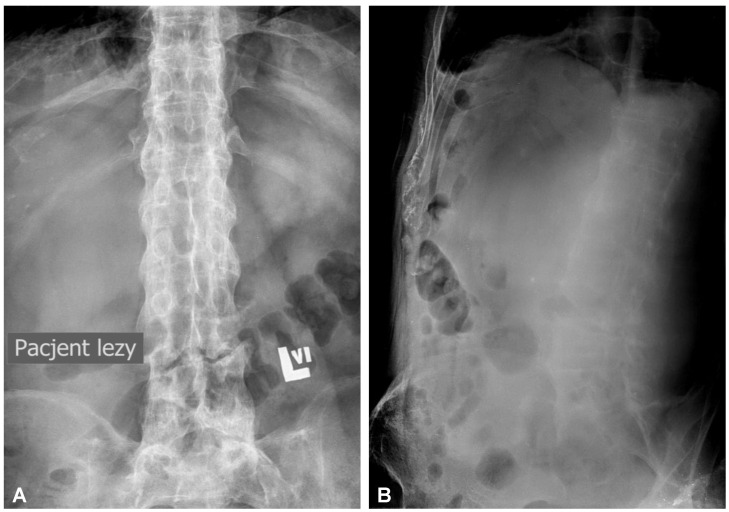
Initial anteroposterior (**A**) and lateral (**B**) plain radiographs of the lumbar spine demonstrating fracture line completely slicing through the L3/L4 intervertebral disc space and features of the extensive calcification and ossification along the vertebral column resembling classic “bamboo spine” formation.

**Figure 2:**
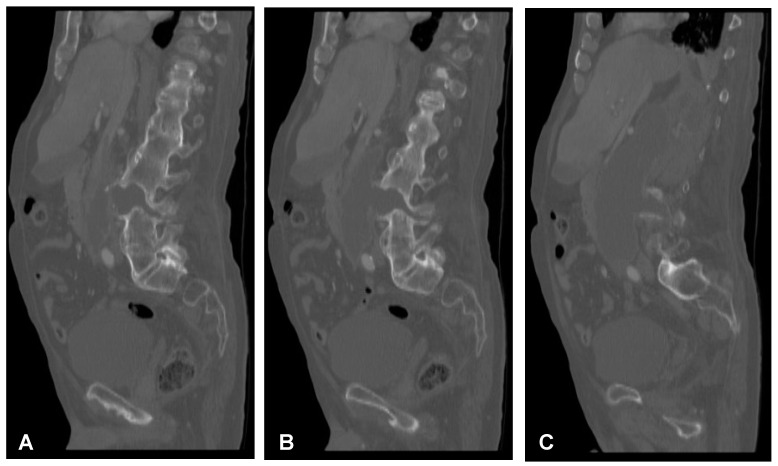

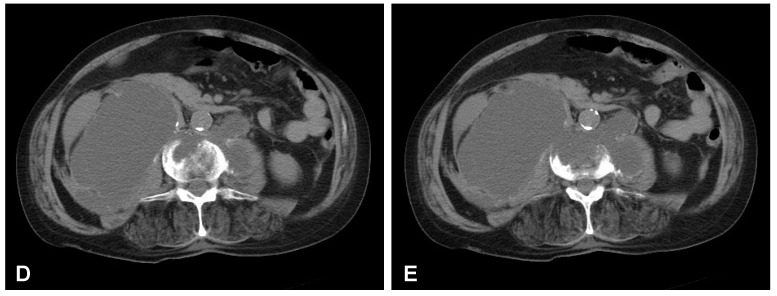
Preoperative sagittal (**A**–**C**) and axial (**D**,**E**) CT scans (non-contrast enhanced) of the lumbar spine demonstrating hypodense area at the L3/L4 intervertebral disc space communicating with both psoas major muscles forming massive well-defined fluid-like collection in the right retroperitoneum.

**Figure 3:**
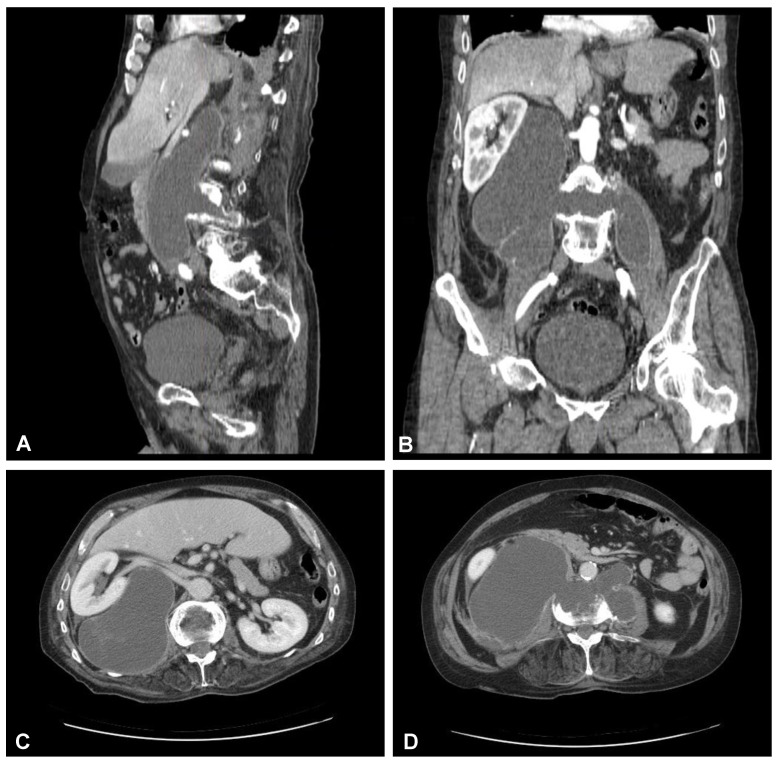
Preoperative sagittal (**A**), coronal (**B**), and axial (**C**,**D**) CT scans (contrast enhanced) of the lumbar spine demonstrating hypodense area with enhancing wall at the L3/L4 intervertebral disc space communicating with both psoas major muscles forming massive well-defined fluid-like collection in the right retroperitoneum.

**Figure 4:**
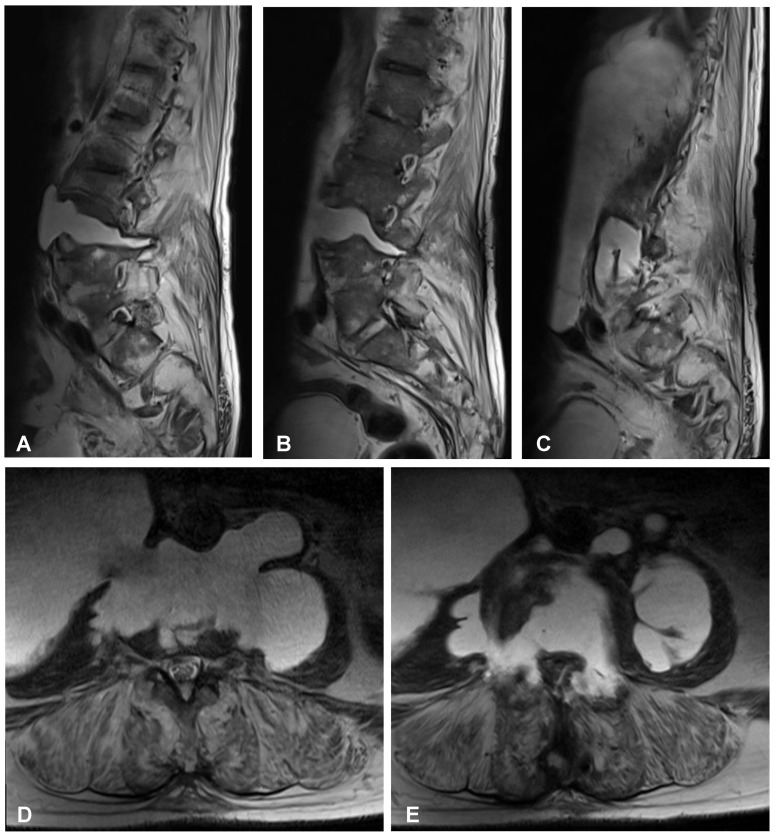
Preoperative sagittal (**A**–**C**) and axial (**D**,**E**) MRI scans (T2-weighted images) of the lumbar spine demonstrating high-intensity area at the L3/L4 intervertebral disc space communicating with both psoas major muscles forming massive well-defined fluid collection in the right retroperitoneum.

A 59-year-old man presented to the hospital with atypical abdominal pain for a few days, three weeks after sustaining a low-energy fall. At the time of admission, the patient had normal upper and lower extremity strength and no pathologic reflexes, as well as no bowel or bladder incontinence. The initial performed plain radiograph of the abdomen demonstrated a fracture line completely slicing through the L3/L4 intervertebral disc space and features of ASH (Figure 1). The additional performed computed tomography (CT) scans demonstrated a three-column fracture of L4 vertebrae with a suspicious transdiscal injury at the L3/L4 level, which was morphologically classified as type B3/C according to the Arbeitsgemeinschaft für Osteosynthesefragen (AO) spine classification. Moreover, at the fracture level, the hypodense area communicating intervertebral disc space with both psoas major muscles forming a massive, well-defined fluid-like collection in the right retroperitoneum was observed (Figure 2). In addition, contrast-enhanced CT (CE-CT) showed a thick enhancing wall and internal septae within an area measuring a total of 10 cm × 20 cm × 10 cm (TR × AP × CC) (Figure 3). Furthermore, the performed magnetic resonance imaging (MRI) scans confirmed the cystic nature of the lesion, which had no specific characteristics and demonstrated intensities that were similar to those of cerebrospinal fluid (CSF) (Figure 4). Taking into consideration the history and radiological features, suspicion of spondylodiscitis with osteomyelitis features and retroperitoneal abscess formation was provided by the radiologist. Blood analysis performed on admission revealed the following results: hemoglobin (Hb): 9.6 g/dL; platelet (PLT): 192 × 10^9^/L; white blood cell (WBC): 9.7 × 10^9^/L; and creatinine (CREA): 0.42 mg/dL. Despite the lack of infection symptoms, the patient was empirically administered intravenous antibiotics, including meropenem (1 g every 8 h) and vanomycin (1 g every 12 h), without waiting for microbiological confirmation.

### 2.2. Surgery of the Lumbar Spine Followed by Nephrectomy and Ureter Ligation

**Figure 5:**
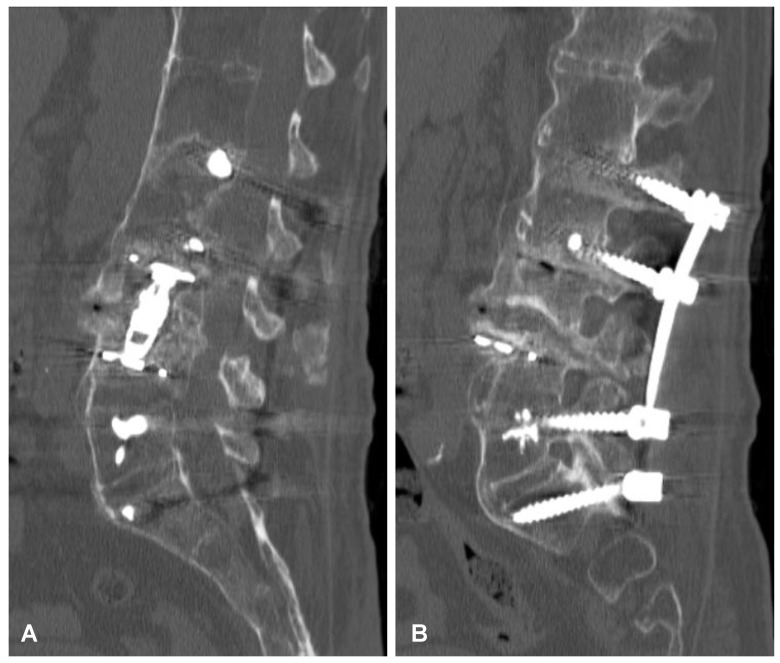
Postoperative sagittal CT scans (non-contrast enhanced) after the two-stage surgery of the lumbar spine demonstrating placement of the distractible vertebral body replacement implant between L3 and L4 (**A**) and posterior percutaneous transpedicular stabilization at the L2-L3-L5-S1 levels (**B**).

**Figure 6:**
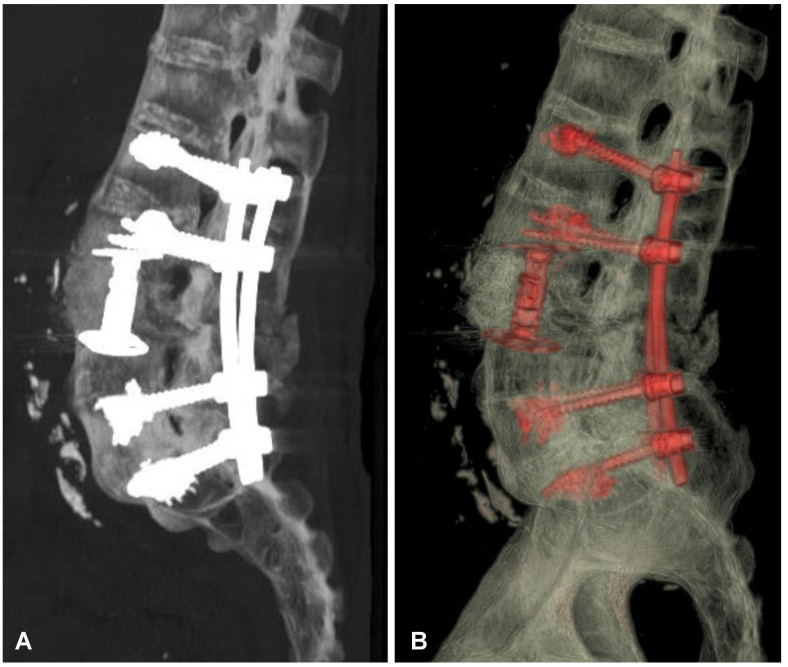
Postoperative CT-based reconstruction of the lumbar spine demonstrating correct positioning of the anterior and posterior spinal implants at the L2-L3-L5-S1 levels (**A**,**B**).

**Figure 7:**
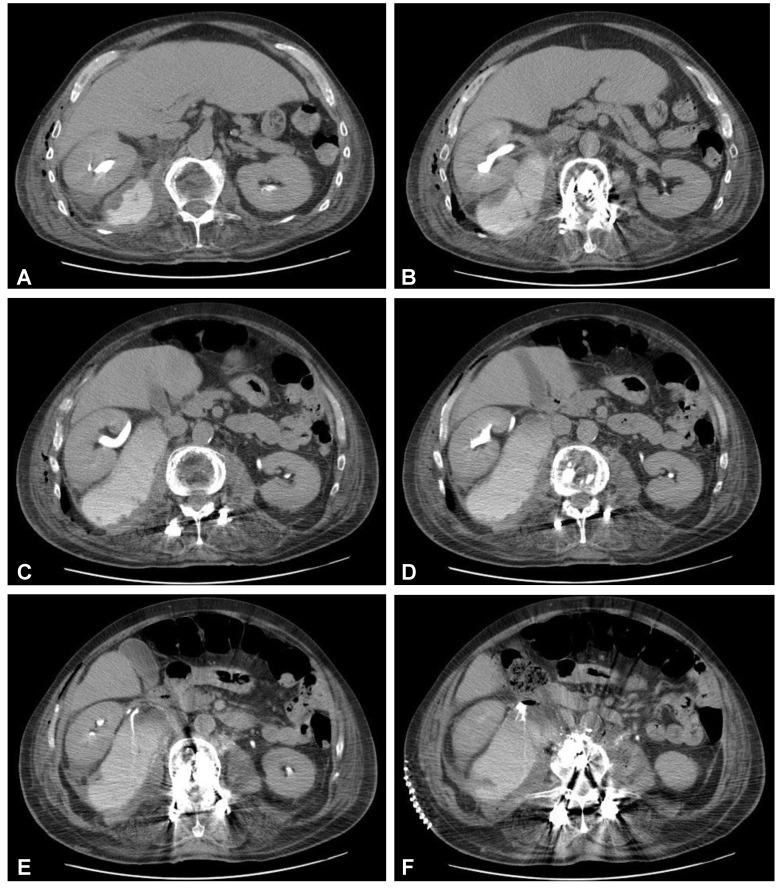
Postoperative axial CT scans (contrast enhanced delayed phase) demonstrating evidence of post-traumatic right ureteral injury, urine leakage, and hydronephroureter (**A**–**F**).

After initial diagnostics and preparations, the patient was transferred to the operating room. The posterior percutaneous transpedicular stabilization at the L2-L3-L5-S1 levels (Mantis; Stryker, Kalamazoo, MI, USA) with cement augmentation (VertaPlex HV; Stryker, Kalamazoo, MI, USA) for each inserted screw (4 mm × 6.5 mm/50 mm; 4 mm × 6.5 mm/45 mm) under intraoperative 2-dimensional (2-D) fluoroscopy control was performed in the first stage of surgery. In the second stage of surgery, from a lateral approach, the left side of the retroperitoneal cyst was emptied, and watery/light yellow fluid similar to urine was obtained. Therefore, a distractible vertebral body replacement implant (AsterX; Medtronic, Minneapolis, MN, USA) was placed between the L3 and L4 bodies with the placement of a retroperitoneal precutaneus Redon drainage system. A control CT scan performed one day after the operation revealed the correct placement of all the implants (Figure 5 and Figure 6). The second CE-CT scan performed two days after surgery consecutively showed evidence of post-traumatic right ureteral injury, urine leakage, and hydronephroureter (Figure 7). The general examination of intraoperatively obtained fluid revealed the following results: pH: 9.0; protein: 600 mg/dL; transparency and color before centrifugation: sanguine and cloudy; transparency and color before centrifugation: transparent and dark yellow; absolute leukocytes (LEU): 1197 cells/μL; and sediment: single round epithelium in the sample with erythrocytes loosely cover the field of vision (15–20 in the counterfield). The microbial culture of the intraoperative obtained fluid after 7 days was negative. After 4 days, the patient was transferred to another local hospital specializing in urological care. Due to the overall late presentation covering advanced hydronephrosis, ureteric stricture formation, and non-functional kidney, the nephrectomy and ureter ligation were performed. After urological treatment, the patient has been on follow-up for 2 years with no evidence of neurological or urological problems.

### 2.3. Clinical Presentation during the Second Treatment Episode

**Figure 8:**
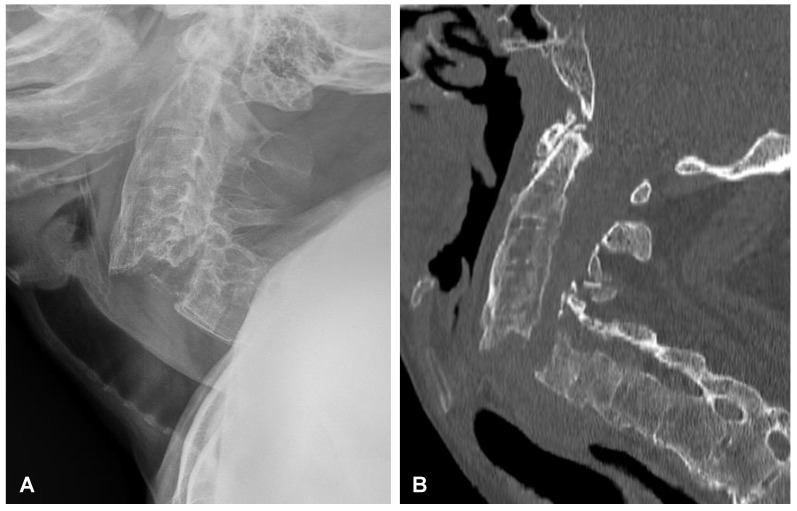
Preoperative plain radiograph (**A**) and CT scan (**B**) of the cervical spine demonstrating unstable extension transdiscal three-column fracture at the C5/C6 level.

Nevertheless, after 2 years of uncomplicated follow-up, the patient presented again to the hospital with tetraparesis after sustaining a low-energy fall. On admission, the patient was conscious, oriented, and hemodynamically stable. During the examination, the neck was stiff and rigid; movements in the lateral direction and flexion/extension were impossible. The initially performed plain radiograph and CT scans of the cervical spine demonstrated an extension transdiscal three-column fracture at the C5/C6 level, which was morphologically classified as type C according to the AO spine classification (Figure 8). Otherwise, there was no evidence of intracranial trauma. Blood analysis performed on admission revealed the following results: Hb: 12.2 g/dL, PLT: 398 × 10^9^/L, WBC: 12.1 × 10^9^/L, CREA: 0.62 mg/dL.

### 2.4. Surgery of the Cervical Spine Due to the Tetraparesis

**Figure 9:**
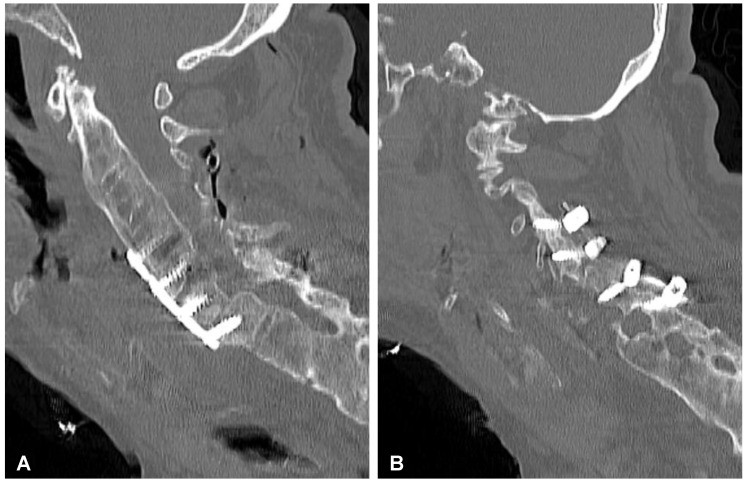
Postoperative sagittal CT scans (non-contrast enhanced) after the two-stage surgery of the cervical spine demonstrating placement of the anterior plate with locking screws (**A**) and posterior lateral mass screws (**B**) at the C4-C5-C6-C7 levels.

Following the initial diagnostics, the patient was transferred to the operating room. The combined anterior and posterior osteosynthesis (360° fixation) at the C4-C5-C6-C7 levels were performed. In the first stage of surgery, the anterior stabilization plate (50 mm) and locking screws (Zephir; Medtronic, Minneapolis, MN, USA) under intraoperative 2-D fluoroscopy control were placed. In the second stage of surgery, the patient was turned and posterior-stabilized (Ellipse; Globus Medical, Audubon, PA, USA) with lateral mass screws (8 mm × 14 mm) using free-hand technique based on anatomical landmarks due to poor intraoperative visualization of cervicothoracic region capabilities using 2-D fluoroscopy was performed. A control CT scan performed one day after the operation revealed the correct placement of all the implants (Figure 9). After surgery, the patient remained in deep tetraparesis with minimal movements in the lower limbs but no movement in the upper limbs. Early postoperative rehabilitation at the bedside was initiated. However, the patient remained prolonged endotracheally intubated but was conscious and awake due to the retention of airway secretions, as well as was parenterally fed for 5 days. The patient was empirically administered intravenously with amoxicillin with clavulanic acid (1 g/0.2 g every 12 h). After successful extubation, the tracheostomy and percutaneous endoscopic gastrostomy (PEG) were not performed. After 12 days, the patient was discharged with no signs of infection and transferred to the rehabilitation center. 

## 3. Discussion

### 3.1. Pathophysiology and Practice Essentials of the Described Disease Entities

Following case reports, an ASH-associated unstable lumbar fracture complicated by ureteral injury mimicking spondylodiscitis was followed by an unstable cervical fracture with tetraparesis in this same patient. ASH constitutes the type of non-inflammatory spondyloarthropathy with a genetic disposition associated with the formation of ectopic calcifications of the ligaments/entheses along the anterolateral part of the vertebral column, leading to bridging of syndesmophytes along the margins of the disc spaces. The precise etiology of ASH is unknown, but several studies have demonstrated its association with advanced age (increased life expectancy), diabetes mellitus, and obesity [9]. In this case, the precise incidence is not known and varies from 2.9 to 25% in different population settings, where it appears to be a 2:1 male predominance, with the prevalence rate in both sexes rising with age and weight [10]. Nevertheless, beware of risk factors; the incidence of this disease seems to be only expected to rise in the coming decades. Therefore, the unstable fractures observed in the ASH course, especially at the cervical and lumbar spine, may be related to the diverse, unusual, but possibly severe and life-threatening complications [11]. Ureteral injury associated with the traumatic event constitutes an especially rare condition, mainly because ureters are situated profoundly in the retroperitoneum and are protected by nearby tissues. In human adults, ureters cover the bilateral fibromuscular tubes that average measure 23.8 cm (±2.18 cm) for men and 23.2 cm (±2.44 cm) for women and are situated anterior to the lumbar vertebral bodies at the L2 and L3 level and psoas major muscle [12]. The anatomical course of the ureters can topologically be divided into three parts, where the proximal portion constitutes a segment that extends from the ureteropelvic junction to the sacroiliac joint, the middle extends portion from the sacroiliac joint to the iliac vessels and the distal portion that extends from the iliac vessels to the bladder. 

### 3.2. Actual State-of-the-Art

To date, only two cases of patients with lumbar fractures and associated ureteral injury have been reported that related to the hyperextension mechanism of injury [7,8]. In the patient reported by us, the initial CT scans demonstrated the predominant presence of spondylodiscitis and bone destruction features, as well as three-column transdiscal fracture at the L3/L4 level, where additional MRI scans demonstrated massive hyperintense fluid collections communicating within this space, which was also confirmed in the surgical field. Considering the overall clinical picture, we supposed that the injury mechanism of our patient depends on hyperextension force caused by the direct impact on the posterior aspect of the lumbar spine. According to the previous case reports and literature regarding the rare possibility of coexistence of vertebral fracture as well as ureteral injury, we supposed that a similar event may have occurred in our case, where potential hyperextension injury of L3 or L4 vertebra with features of ASH due to the rigid nature of the spine may have resulted in stretching ureteral injury. The clinical profile of our patient was dominated by the presence of massive fluid collections that were penetrating the L3/L4 intervertebral space, which for us was mimicking spondylodiscitis and osteomyelitis features with accompanying retroperitoneal massive abscess formation. In this case, despite the lack of infection symptoms and uncertain diagnosis, in supporting patient safety, the wide board-spectrum antibiotics were administered. Ultimately, the infectious component regarding bacterial spondylodiscitis and osteomyelitis was excluded, and the patient in this case did not suffer from urinary tract infection or asymptomatic bacteriuria. 

### 3.3. Clinical Recommendations and Practical Guidelines

Taking into account the increased life expectancy and its associated possible increased number of lumbar spine fractures with ASH-like features, consideration of the presence of potential complications such as ureteral stretching, entrapment injury, or other retroperitoneal structures injuries should be recommended in the diagnostic algorithm. The patient on admission presented no prominent symptoms or signs of ureter injury, including hematuria, dysuria, anuria, or voiding difficulty. Nevertheless, in this case, we have unfortunately diagnosed this state first after a second postoperative CT scan. After all, the ureter injury was missed, and due to the overall late presentation potentially covering weeks, the renal unit was lost. Therefore, the ureter injury prognosis is better when damage is detected as soon as possible and immediate repair is performed [13]. It was observed that delayed diagnosis is associated with a higher complication rate, including the occurrence of urinoma, fistula, and infection, as well as higher reanastomosis failed ratio, increased nephrectomy rate, and prolonged hospitalization. In this case, different management options can be considered depending on the level of injury and its nature. Nevertheless, even with a far-reaching injury, ureter injury does not reveal notable symptoms initially, and its diagnosis is usually delayed [14]. Unfortunately, after 2 years of recovery, this same patient presented again with an unstable three-column fracture at the C5/C6 level and tetraparesis. In this case, we observed a classic ASH-associated extension cervical fracture, which makes up 75% of fractures in this patient population [15]. Furthermore, our patient presented a significant neurological deficit that is also consistent with the high rate of spinal cord injury (SCI) in this patient population [16]. Therefore, according to the literature, the mortality rate in ASH patients with displaced cervical spine fractures has been as high as 35%. This rate is correlated with the patient’s age, the number of comorbidities, and low-energy trauma [17]. In our case, combined circumferential anterior and posterior instrumentation was performed initially due to the highly compromised structural integrity of the vertebral body, kyphotic fracture location, presumed poor bone quality, and expected difficulty in localizing anatomical landmarks. Additionally, our patient’s condition was further complicated by the prolonged endotracheal intubation and the development of pneumonia. Noteworthy, according to the literature, pulmonary complications are commonly seen in ASH patients, where the lungs are frequently fibrotic and the ribs ankylosed, causing rigid rib cage that also, in some cases, could interfere with the surgical strategy [18,19].

## 4. Conclusions

Hereby, we report an unusual and rare case of a patient with ASH and a lumbar spine fracture complicated by ureteral injury mimicking spondylodiscitis with osteomyelitis features and abscess formation, followed by the cervical spine fracture. According to our advanced literature search, to date, no similar case with an identical complicated clinical constellation has been described in the literature. Although the presented case is extremely rare, the potential injury of retroperitoneal structures, including the ureter, after hyperextensive lumbar spine fracture should be considered in ASH patients. Despite the early symptomatology of ureter injury being nonspecific, an immediate diagnosis is crucial in order to avoid complications covering sepsis or potential loss of the renal unit. Furthermore, the atypical clinical presentation regarding the observed spondylodiscitis and osteomyelitis-like features in this case could represent a formidable diagnostic challenge. Based on these findings, contrast-enhanced CT scan of the urinary tract should be considered in outlining the topo-anatomical features of the injured ureter. Therefore, regarding the complex biomechanical changes related to ASH-affected spine, the appropriate high-quality radiographic evaluation involving the entire spinal axis should be recommended in diagnostic algorithms even after low-energy trauma. In this case, treatment is more difficult than in the non-ossified spine and requires appropriate surgical planning to address proper fracture reduction. Collectively, our case emphasizes the importance of awareness and early diagnosis when retroperitoneal structures, including the ureter, occur after a spine fracture, which requires multidisciplinary management and defining long-term care. 

## Data Availability

No new data were created or analyzed in this study. Data sharing is not applicable to this article.

## References

[1] Whang P.G., Goldberg G., Lawrence J.P., Hong J., Harrop J.S., Anderson D.G., Albert T.J., Vaccaro A.R. (2009). The management of spinal injuries in patients with ankylosing spondylitis or diffuse idiopathic skeletal hyperostosis: A comparison of treatment methods and clinical outcomes. J. Spinal Disord. Tech..

[2] McGonagle D., Emery P. (2000). Enthesitis, osteitis, microbes, biomechanics, and immune reactivity in ankylosing spondylitis. J. Rheumatol..

[3] Westerveld L.A., van Bemmel J.C., Dhert W.J., Oner F.C., Verlaan J.J. (2014). Clinical outcome after traumatic spinal fractures in patients with ankylosing spinal disorders compared with control patients. Spine J..

[4] Westerveld L.A., Verlaan J.J., Oner F.C. (2009). Spinal fractures in patients with ankylosing spinal disorders: A systematic review of the literature on treatment, neurological status and complications. Eur. Spine J..

[5] Pereira B.M., Ogilvie M.P., Gomez-Rodriguez J.C., Ryan M.L., Peña D., Marttos A.C., Pizano L.R., McKenney M.G. (2010). A review of ureteral injuries after external trauma. Scand. J. Trauma. Resusc. Emerg. Med..

[6] Siram S.M., Gerald S.Z., Greene W.R., Hughes K., Oyetunji T.A., Chrouser K., Cornwell E.E., Chang D.C. (2010). Ureteral trauma: Patterns and mechanisms of injury of an uncommon condition. Am. J. Surg..

[7] Kawasaki S., Shigematsu H., Matsumori H., Maegawa N., Tanaka Y. (2018). Ureteral injury as a possible complication of vertebral fracture in a patient with ankylosing spinal hyperostosis. J. Orthop. Sci..

[8] Oh I.S., Chang D.G., Kim Y.H., Ha K.Y. (2013). Pure hyperextension injury of the lower lumbar spine with an ureteral impingement. Eur. Spine J..

[9] Vaishya R., Vijay V., Nwagbara I.C., Agarwal A.K. (2017). Diffuse idiopathic skeletal hyperostosis (DISH)—A common but less known cause of back pain. J. Clin. Orthop. Trauma.

[10] Fornasier V.L., Littlejohn G., Urowitz M.B., Keystone E.C., Smythe H.A. (1983). Spinal entheseal new bone formation: The early changes of spinal diffuse idiopathic skeletal hyperostosis. J. Rheumatol..

[11] Romero-Muñoz L.M., Tipper G., Segura-Fragoso A., Barriga-Martín A. (2022). Outcomes of spinal cord injury following cervical fracture in ankylosing spondylitis and diffuse idiopathic skeletal hyperostosis (DISH): A prospective cohort study. Neurocirugia (Astur. Engl. Ed.).

[12] Mansouri A., Tostivint V., Rouvellat P., Roumiguié M., Gamé X., Huyghe E., Rischmann P., Thanwerdas J., Malavaud P. (2019). La longueur de l’uretère est-elle liée à la taille du patient? [Is the ureteral length associated with the patient’s size?]. Prog. Urol..

[13] Kominsky H.D., Shah N.C., Beecroft N.J., Diab D., Crescenze I.M., Posid T., Baradaran N. (2021). Does Timing of Diagnosis and Management of Iatrogenic Ureter Injuries Affect Outcomes? Experience From a Tertiary Center. Urology.

[14] Lai C.J., Chang M.Y., Huang P.C., Chu Y.C. (2019). Complete ureter avulsion causing a long defect as a complication of posterior spine fusion: A rare case treated with nonrobotic laparoscopic repair. Res. Rep. Urol..

[15] Olerud C., Frost A., Bring J. (1996). Spinal fractures in patients with ankylosing spondylitis. Eur. Spine J..

[16] Fox M.W., Onofrio B.M., Kilgore J.E. (1993). Neurological complications of ankylosing spondylitis. J. Neurosurg..

[17] Taggard D.A., Traynelis V.C. (2000). Management of cervical spinal fractures in ankylosing spondylitis with posterior fixation. Spine.

[18] Lazennec J.Y., d’Astorg H., Rousseau M.A. (2015). Cervical spine surgery in ankylosing spondylitis: Review and current concept. Orthop. Traumatol. Surg. Res..

[19] Samartzis D., Anderson D.G., Shen F.H. (2005). Multiple and simultaneous spine fractures in ankylosing spondylitis: Case report. Spine.

